# How new plant species have been discovered in China: collection gaps and preferences over the past century

**DOI:** 10.3389/fpls.2025.1605431

**Published:** 2025-07-10

**Authors:** Dongmin Shi, Kuiling Zu, Jiahui Nong, Wenjing Yang, Yuting Zhang, Shuai Liao, Guojin Zhu, Jie Sun

**Affiliations:** ^1^ Jiangxi Key Laboratory of Subtropical Forest Resources Cultivation, College of Forestry, Jiangxi Agricultural University, Nanchang, China; ^2^ Grassland Administration on Forest Ecosystem Protection and Restoration of Poyang Lake Watershed and Jiangxi Provincial Key Laboratory of Conservation Biology, College of Forestry, Jiangxi Agricultural University, Nanchang, China; ^3^ Key Laboratory of Poyang Lake Wetland and Watershed Research (Jiangxi Normal University), Ministry of Education, Nanchang, China; ^4^ State Key Laboratory of Plant Diversity and Specialty Crops, South China Botanical Garden, Chinese Academy of Sciences, Guangzhou, China

**Keywords:** biodiversity, digitized plant specimen, herbarium, specimen collection, taxonomic practice, plant conservation

## Abstract

**Introduction:**

How many species there are in the world remains a fundamental scientific question, serving as a critical reference for formulating and implementing effective biodiversity conservation strategies. The accelerating global biodiversity crisis has propelled scientific interest in understanding spatial-temporal patterns of new species discovery, particularly as these findings inform urgent conservation priorities. Digitalization of plant specimens provides important information on the discovery process of new species and clarifies the general situation of the core distributions in China over the last century. However, the new species discovery process of plants has not been the focus of much attention in Asia. The study analyzes digitized herbarium specimen data to investigate new species discoveries, specimen collection gaps, and collection preferences in China over the past century.

**Methods:**

First, we collected the herbarium type specimens data. Then we analyzed sampling biases of functional traits (life form, floral colors, fruit colors and types) in specimens collection and summarize distributional patterns in new species discovery. We answered the question of what plant species are more likely to be found and explored the distribution areas of new species discoveries over the last century.

**Results:**

Our results reveal that there are more new plant species been found in herbaceous plants (90.8%) than woody (9.2%) plants, and new species with bright flowers and fruits, and the smaller height are easier to be discovered. There is also an observable inter-annual and seasonal variation in the discovery of new species, with peak collections occurring four year periods, and summer (June-August) witnessing the highest number of new species discoveries. The southern regions of China offer easier access to new species, and higher numbers of new species have been identified in the regions with the richness of plant diversity. The number of specimens collected by individuals (58%) surpasses those collected by teams (42%).

**Discussion:**

More attention should also be paid to species with different plant functional traits (herbs, species with bright flowers and fruits) and the potential distribution of vacant regions. This study provides scientific reference and direction for the accelerating discovery of more new species in the future, and also contribute to the future conservation of biodiversity.

## 1 Introduction

How many plant species are in the world is a fundamental scientific question that researchers in the field of conservation biology need to answer (Pimm and Joppa, 2015). There are 996,093 at species level and 342,953 accepted vascular plant species based on the World Checklist of Vascular Plants (WCVP) (Govaerts et al., 2021). Approximately 80% biomass is provided by plants on earth (Bar-On et al., 2018), and plants play an indispensable role in maintaining the dynamic balance of biological communities, preserving genetic diversity and ensuring the proper functioning of ecosystem services (Wan and Zhang, 2021). However, our understanding of global biodiversity remains limited, and the exploration of the plant kingdom itself is far from exhaustive. Nevertheless, scientists predict that there are still many new plant species to be discovered in the future (Ondo et al., 2024; Joppa et al., 2011). The discovery of new species is of great importance for biodiversity conservation, and helps to assist in assessing the status of threatened species and predicting potential risks of these species (Malhi et al., 2020). Furthermore, the study of the history of new species discovery helps to facilitate its progress in the future.

Herbaria serve as repositories to safeguard specimens for an extended duration and provide support for academic research (Davis, 2023; Lang et al., 2018). There are 480 million herbarium specimens across the world through the efforts of thousands of botanists for over four centuries (Heberling and Isaac, 2017). There are more than 16 million specimens in China’s herbaria (Yang, 2013), which are important physical materials for studies of botany and biodiversity science (Panchen et al., 2019). Herbarium specimens are essential for expanding taxon or population sampling, preserving rare or even extinct species (Welch et al., 2016). Additionally, herbarium specimens carry important information regarding collection date, geography distribution, plant functional traits and genetic material information (Eckert et al., 2024; Marín-Rodulfo et al., 2024). Notably, type specimens serve as the inaugural vouchers for newly described plant species, recording of which indicate the distribution, traits, collect times and collectors of the new species (Jang et al., 2022). Although China maintains extensive plant specimen holdings, its type specimens are disproportionately scarce relative to global standards (Yang, 2013). The type specimens of plant species provided information for the research on the discovery process of new species, therefore, the information obtained from them is different than that of conventional herbarium specimens.

With the development of online technology, digitized herbaria not only help the management, retrieval, utilization and protection of specimens, but also provide a new way for the biodiversity discovery and conservation (Davis, 2023; Wu et al., 2024). For example, digitized plant specimens provide historical flowering and fruiting time that could be used to study phenological change driven by climate (Zu et al., 2023a; Park et al., 2023; Ramirez-Parada et al., 2022). Digitalizing plant specimens provide the possibility of getting functional traits and geographical data from global collections and access to Chinese plant specimens from overseas (Daru, 2025). This process is an invaluable resource for understanding plant geographical distribution and for the conservation of plant communities (Rocchetti et al., 2021). For example, a recent study (Daru, 2025), used digitized specimen databases (e.g. GBIF, iDigBio and Flickr), to create machine learning models that fill in plant trait gaps and conduct global plant shape biodiversity research. Natural history collections, containing specimens across extensive time and geographical ranges, are also help to understand the biological invasion dynamics (Holmes et al., 2016; Ni, 2022). The interactions between plants and the insect herbivores altered by the environmental temperature and urbanization have been detected based on the herbarium specimens during the past century (Meineke et al., 2018). However, few studies have used digitized records of historical herbarium collections to explore the rule of discovery of new plant species, which is of great significance for the further discovery of new species and the conservation of regional biodiversity.

Previous studies of the discovery of new plant species have focused mainly on what kinds of species are more likely to be found (Adamo et al., 2021), and who will describe new plant species (Liu et al., 2023). However, how the new plant species have been discovered during the past century has received limited attention. Most plant specimens only provide information such as location, collection date and habitat (Willis et al., 2017). A new study suggested that the new plant species have narrowed distributional range size and smaller population sizes than the already known species, and the new finding species tends to be located in a specific geographic space or a endemic species (Cheek et al., 2020). Generally, plant specimen collection preferences are affected by many factors, including species traits, socio-economic factors, geographical and environmental factors (Zhang et al., 2024a). Furthermore botanists and ecologists often pay more attention to species in the regions that are easily accessible or close to their research facility (Moerman and Estabrook, 2006). Previous specimen collection primarily relied on knowledgeable individuals, whose personal preferences and interests may result in bias on the collection process (Küper et al., 2006). Therefore, the discovery of new species are impacted by many factors including the functional traits of the plants, the collectors and other factors. A study of the history of new species discovery can shed light on the determinants that influence the discovery of new plant species and implications for future plant species discoveries and descriptions.

China has one of the richest national floras and is the most plant diverse country in the northern hemisphere, including four global biodiversity hotspots and approximately 33,000 angiosperms plants (Yang et al., 2013). However, nearly half of all angiosperms in China are endemic, including a total of 15,009 species (45.47%), belonging to 183 families, 1,489 genera (Huang et al., 2014). Due to climate change, human activities (e.g. urbanization construction, industrial pollution) and biological invasion, China has become the most threatened country in terms of biodiversity, and many rare and endangered endemic angiosperms are in urgent need of protection (Chen et al., 2024). Fortunately, millions of plant specimens have been collected and stored in herbaria over the past century (Yang et al., 2013). A wide variety of plant specimen digitization was started since twenty years ago in the majority of Chinese herbaria including most type specimens. These digitized specimens provide important data for the study of the history of the discovery of new species of plants.

In this study, we used the digitized type specimens from the Chinese herbaria, combining with the plant functional traits data, this paper explores the collection gaps and plant functional trait preferences of new species in China over the past century. We aim to answer the following questions: (1) What are the most common functional traits found in the newly described species? (2) Are there geographical and temporal gaps in the discovery of new plant species? (3) How did the collectors contribute to the discovery of new species? This study could provide new research directions and ideas for the using of the digitized specimens and its importance for biodiversity conservation.

## 2 Materials and methods

### 2.1 The new species data

The digitized type specimens of the new species were obtained from the National Specimen Information Infrastructure (NSII, http://www.nsii.org.cn/), the Chinese Plant Names Index (CPNI, https://cpni.ibiodiversity.net/), and the Chinese Virtual Herbarium (CVH, https://www.cvh.ac.cn/). In total, 26,594 herbarium type specimens (e.g. holotype, lectotype, epitype, neotype, syntype, isotype) were extracted for all plant species since 1900. Among them, most of the new species are primarily based on holotype specimens. The type specimens include the complete information on scientific names, locations, collected data, and collectors. These digitized type specimens were from a total of 36 large herbaria in China, among which the top ten herbaria with the largest distribution of specimens are shown in **Supplementary Figure S1**.

### 2.2 Data cleaning

In order to ensure the quality of the data, we have cleaned the data of the plant species’ type specimens. Firstly, we have standardized data processing, including uniform treatment of the of names of the collectors and the description of species’ distribution sites. The specimens lacking coordinates were georeferenced using Google Earth. We also categorized collectors into teams and individuals, and treating more than two collectors as teams. Secondly, we have comprehensively cleaned up the collection data, including the year, month and date, unified the time expression format and the errors. And we disproportionately excluded older specimens without accounting for differences in preservation time and recording standards. Besides, we standardized the species’ scientific names of all specimens based on the Catalogue of Life (COL, http://www.catalogueoflife.org/) We excluded the duplicate specimens lacking information on the distributional locations, and only included records with full information, including the collection year, collectors, and plants’ names. Finally, a total of 16121 specimens were used in the following analyses.

### 2.3 Plant traits data

In order to explore what functional traits of species are more likely to be collected as a new species during the historical period, the functional traits of different species were statistically analyzed in this study. We classified the species according to whether they are threatened plants and whether they are endemic to China. We also counted the life forms, flower colour, fruit colour and fruit types of the different species. Following previous research indicating that plant specimens with showy flower and fruit colors are more likely to be collected (Zhang et al., 2024a), we categorized flower colours as white, yellow, purple, red, etc., and fruit colours as red, brown, purple, black, etc, and regarding the plant functional traits recorded in Species 2000 (http://sp2000.org.cn), we classified the growth form of new species specimens as herbaceous or woody, and the fruit type as capsule, berry, drupe, etc, to explore the potential bias in new species discovery. The threatened plants were reference the threatened Species List of China’s Higher Plants, and we divided them as Critically Endangered (CR), Endangered (EN) and Vulnerable (VU) species (Qin et al., 2017). The endemic species refer to the species diversity that naturally exists with China’s geographical region (Huang et al., 2014). The life forms, flower colour, fruit colour and fruit types according to Flora of China (FOC, http://www.iplant.cn/foc), Flora Reipublicae Popularis Sinicae (FRPS, http://www.iplant.cn/frps).

### 2.4 Data analysis

To explore what kinds of species are likely to be identified as a new species, we calculated the number of specimens collections and the species traits. Specifically, we counted the top 10 families and genera with the largest proportion of species and specimens, and we counted the proportion of endemic, non-endemic, and endangered species among these new species. Moreover, for exploring what traits of plants are more likely to be collected as new species, we also counted the proportion of different life forms flower colour, fruit colour and fruit types in the new species.

In order to investigate what types of new species are more likely to be discovered, we explored the time preference of new species discovery over the entire time period from 1900 to 2022, and we also compared the monthly deviation of new species collection in different months on the inter-annual scale. To understand where the discovery of new species in China is concentrated, we count the number of occurrences in each province and each city/county. Then we converted these counts into image information for visualization on the map of China. Also, we took 500 m as an interval for grouping and analyzed the elevation preference of each new species. Then we counted the number of species specimens and species number in each elevation interval, and explored the distribution pattern of new species at different elevational belts. In order to explore whether the discovery of new species is related to regional species diversity, the linear regression method was used in this study to explore the relationship between the number of new species and regional species diversity. The data of regional species diversity was obtained from China Checklist of Higher Plants (Liu and Qin, 2023).

**Figure 1:**
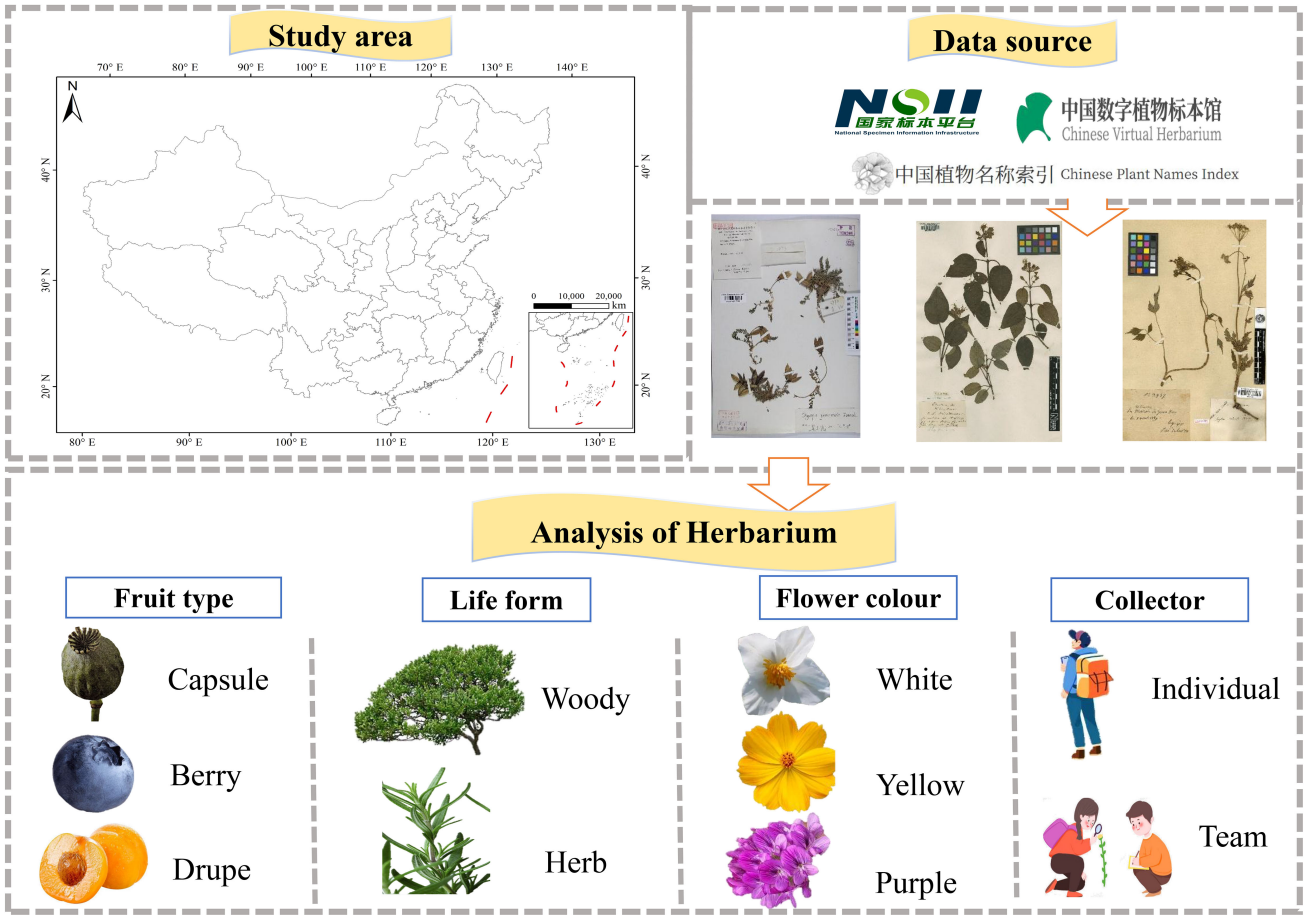
Flowcharts for the discovery of new species using specimen. The specimen images are from (https://www.cvh.ac.cn), and the other images are from the internet (https://www.flaticon.com).

Finally, we further analyzed the preferences of the discoverers of new species to answer the question of who collected the new species. We measured who discovered more new species between team and individual collectors, and the regularity of team and individual discovery of new species over time. We also calculated the top five teams and individuals that collected the most. Above all, the covered research scope, data sources, data cleaning, and data analysis processes are clearly presented (**Figure 1**). Each component is closely interlinked, providing a systematic and coherent framework that supports the orderly progression of the research.

## 3 Results

### 3.1 Plant taxa preferences

A total of 8734 new Chinese species have been described, belonging to1455 genera and 251 families and there was an average of six duplicate specimens per new species. Among them, 259 (2.97%) endangered species and 1006 (11.52%) endemic species were recorded. The largest number of new plant species are non-endemic, accounting 88.4% of the total (**Table 1**). Among the top 30 families with the highest number of new species discovered, Orchidaceae is the largest (637 species/744 specimens), followed by Rosaceae with 530 species (2718 specimens). Among the top 30 genus, *Elatostema* is the largest (270 species, 281 specimens), followed by *Carex* which is 180 species (199 specimens) (**Supplementary Figure S2**). As different genera/families of plants have different fruit types, we found that the proportion of newly discovered species with capsule fruits was the largest, which is 545 species (28.7%), followed by the species with berry (13.3%), species with drupe (13.2%), and species with achenes (11.2%) (**Supplementary Figure S3**).

**Table 1:** The number of species, genus and family of different species groups (endangered species, endemic species, and non-endemic species).

Type	Number of species	Family	Genus
Endangered species	259	72	157
Endemic species	1006	96	303
Non-endemic species	7728	155	1152
All	8734	251	1455

**Figure 2:**
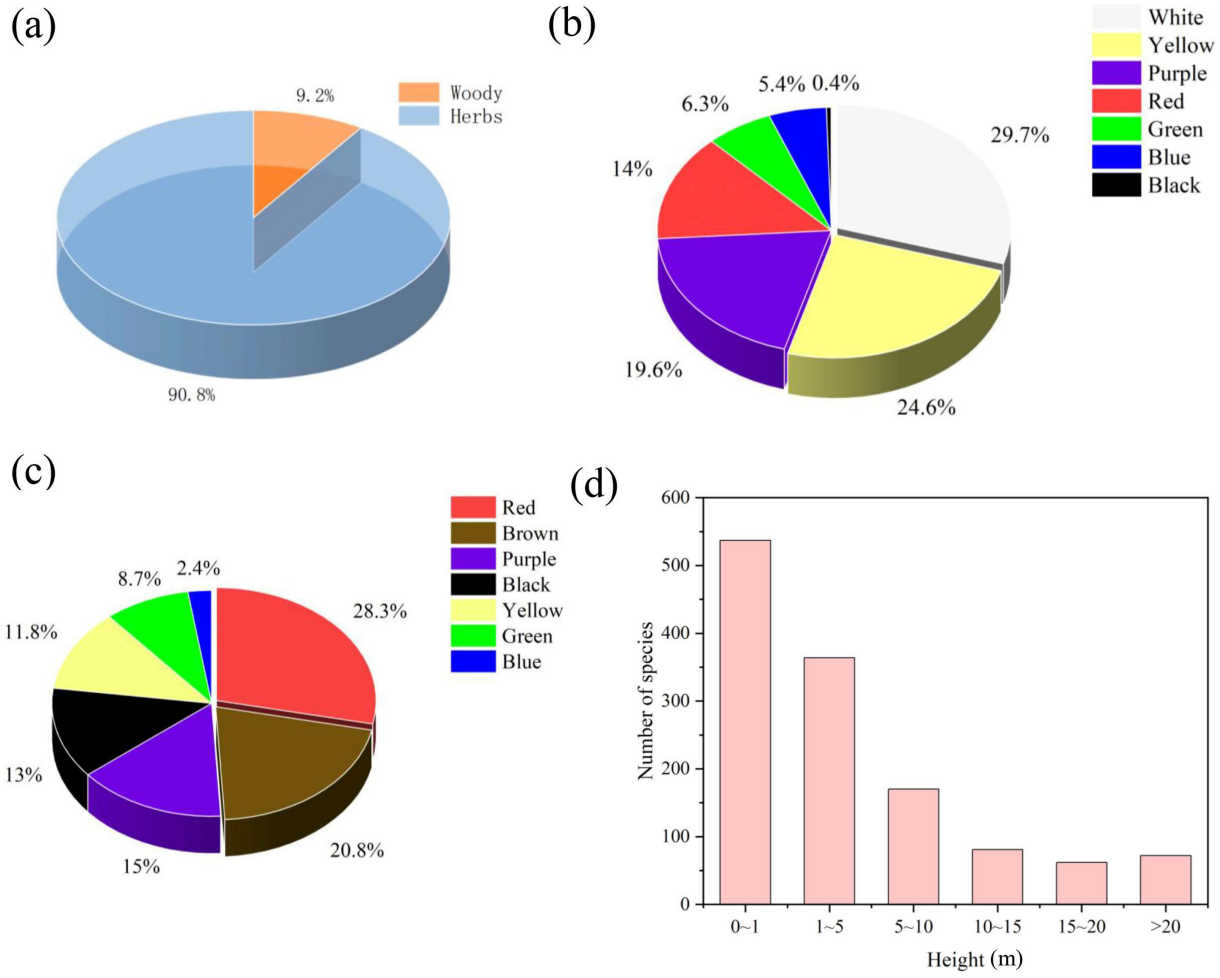
The proportion of new species in herbaceous and woody plants **(a)**, different flower colour **(b)**, fruit color types **(c)** and maximum plant height **(d)**.

There are 7930 (90.8%) herbaceous species and 904 woody species (9.2%) in the new plant species (**Figure 2a**). Among woody plants, the proportion of plants with drupe fruit type was the highest, accounting for 29% (209 species). Among herbaceous species, the proportion of plants with capsule fruit type was the highest, accounting for 37.8% (444 species) (**Supplementary Figure S4**). We found that the proportion of plants with white-coloured flowers was the highest, accounting for 29.7%, following by the species with yellow-coloured flowers (24.6%) and purple flowers (19.6%) (**Figure 2b**). Similarly, the proportion of plants with red-coloured fruits was the highest, accounting for 28.3%, following by the species with brown-coloured fruits (20.8%) and purple fruits (15%) (**Figure 2c**). Conversely, the new plant species with blue and green flowers/fruits were collected less. With the increase of plant height, the number of new species discovered gradually decreased (**Figure 2d**).

### 3.2 Time and geographical distribution biases

**Figure 3:**
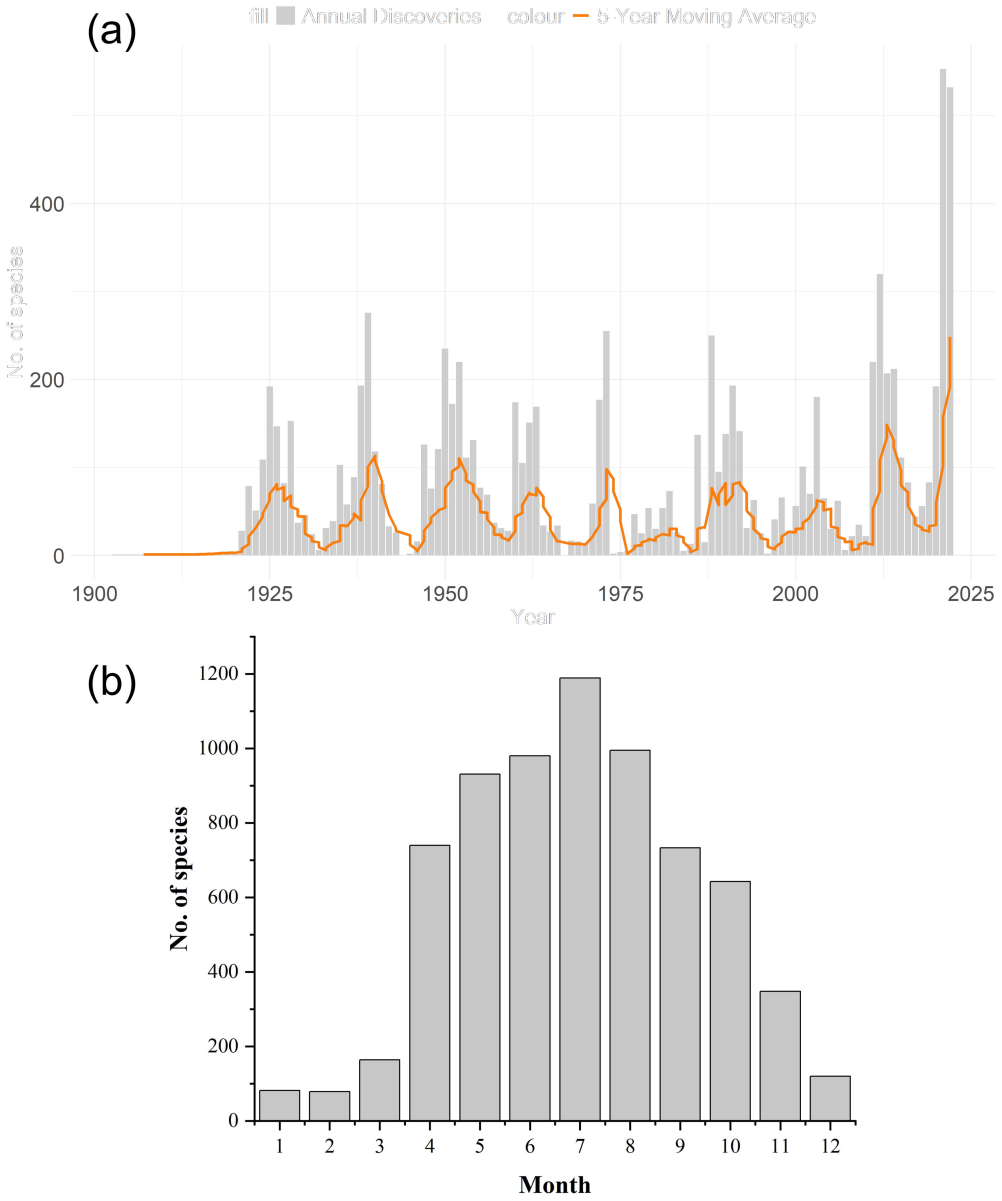
The number of species of newly discovered plants changed by year **(a)** and month **(b)**.

During the past century, there are four peaks (1920 - 1940, 1950 - 1960, 1970 - 1990 and 2000 - 2022) in the number of new species discovered. From January to July, the number of newly discovered species increased and reached its peak in July, and then gradually decreased from July to December. The highest number of new species are discovered in summer, which is also the growing season for plants (**Figure 3**).

**Figure 4:**
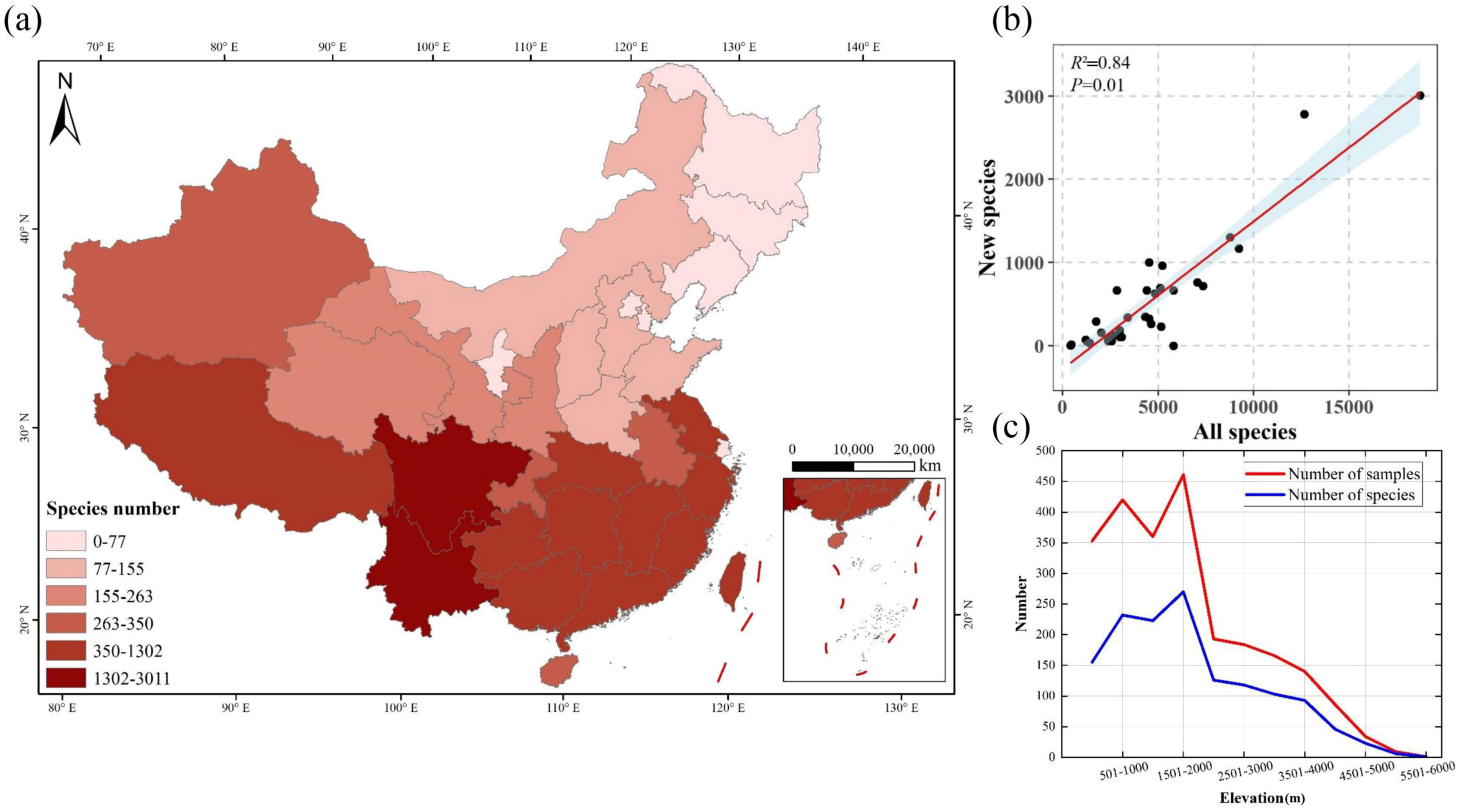
Geographic distribution in herbarium collection of new plant species **(a)**, relationship between the number of new species and the total number of species in each province **(b)**, and the number of new species along the elevational gradient **(c)**.

The number of new species in the south is higher than that in the north of China, and Sichuan and Yunnan provinces had been found the highest number of new species (**Figure 4a**). The regional species diversity was positively correlated with the number of new species (*R* ^2^ = 0.84, *P* = 0.01) (**Figure 4b**). The counties/cities with the highest incidence of newly discovered species are predominantly Wenchuan, where the ratio of new species to the total species count stands at 2.51%. This is followed closely by Nanchong (1.72%), Nanjing (1.42%), Motuo (0.89%), and Lichuan (0.88%) (**Supplementary Table S1**). Our results also show that the number of new species found varies at different elevational belts, and the number of new species increased first and then decreased along the increase of altitude. The highest number of new species was found at the interval of 1501 - 2000 m altitude (**Figure 4c**).

### 3.3 The contribution of collectors to the discovery of new species

**Figure 5:**
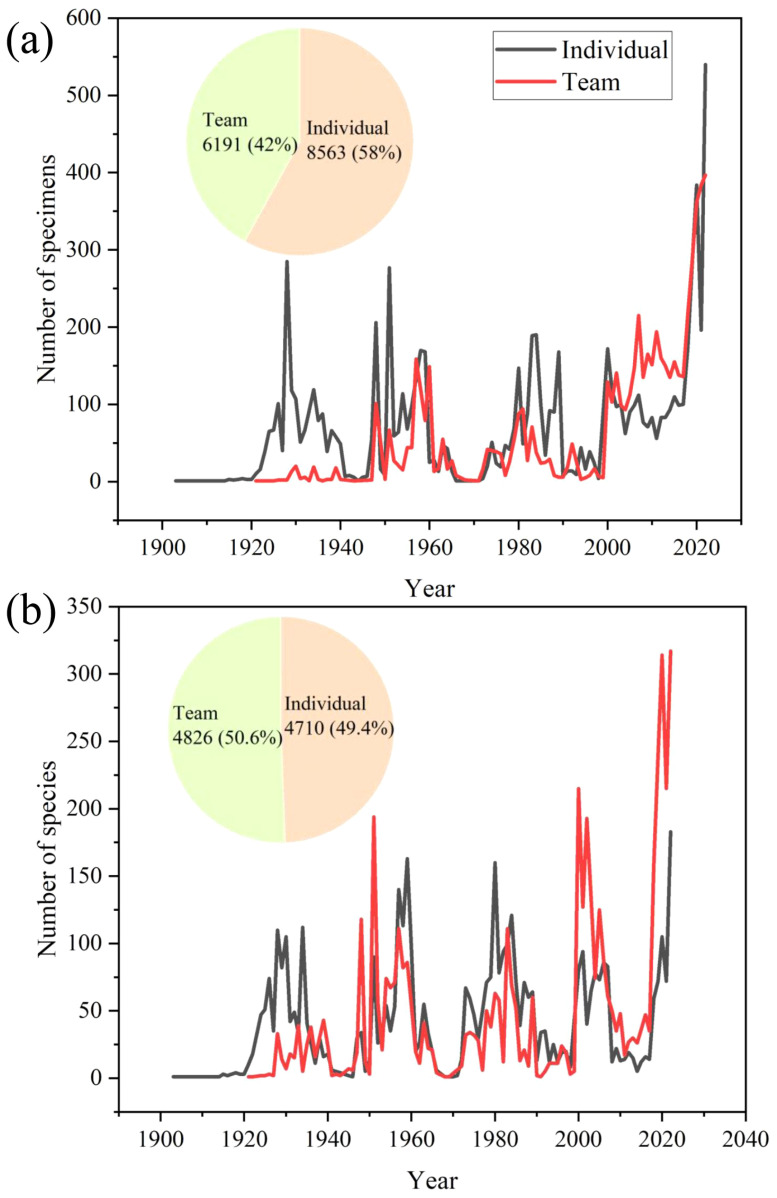
The number of new species specimens discovered by teams and individual collectors **(a)** and the number of species discovered by teams and individual collectors **(b)** during the past century.

Among the number of collected specimens of new species, there are some differences between the ones collected by teams and individuals. Since more specimens of new species are collected by individuals (58%), and less by collection teams 230 (42%). There was little difference between the contribution of individuals and teams to the number of new species collected. However, in the last two decades, the team has contributed significantly to the discovery of new species (**Figure 5**).

## 4 Discussion

In this study, we extracted 26,594 specimens, various hypotheses about formation of Chinese new specimen collection gaps and preferences over the past century have tested. There are significant correlations between the number of new species discovered and plant functional trait factors (including life form, flower color and fruit type). The time and geographical range of specimen collection have an impact on the number of new species discovered. In addition, collectors’ preferences also influence the discovery of new species. We recommend that future specimen collection efforts should be put into exploring high-altitude and mid-high-latitude areas with lower species richness, and on plants with small, dull-colored flowers and fruits across different taxa, with the goal of enhancing the potential for identifying more new species.

### 4.1 Taxa gaps and preferences of new plant species

According to our analysis, there are significant differences among the newly discovered plants species in different taxonomic categories, and the species with specific traits (e.g. herbs, species with bright flowers and fruits) are more likely to be collected and identified as new species. Our results are similar to previous study that the colorful, conspicuous and broadly distributed flowers are more easy to be collected (Adamo et al., 2021). However, our results indicated that certain taxa are easier to be found in the discovery of new species. One reason is that botanists tend to specialize and therefore focus on one or more plant groups, which often results in more frequent collection of new species within specific groups (Bebber et al., 2010). For instance, biological collecting biases existed in the plants with green or brown inflorescences, and very small plants (Schmidt-Lebuhn et al., 2013). Another reason is that, while most new species are described, identified, and classified in herbaria, approximately 50% of tropical plant specimens are misidentified due to the complexity of biodiversity (Davis, 2023). Previous studies conclude that there are nearly 20% unnamed species in the herbaria, and these species are misidentified or undescribed by the taxonomic experts (Joppa et al., 2010). If unnamed specimens remain unrecognized, it would seriously underestimate the taxonomic bias of new species discoveries. Therefore, the finding (including the naming) of new species is crucial for scientific research. Without scientific naming, the likelihood of conducting biodiversity research, assessing the endangered status of species, and implementing scientific conservation measures would be reduced (Wan and Zhang, 2021).

Besides, we also found that some of these new species are endangered and endemic. For instance, nearly 70% plants are endemic, with thousands of plant species remaining to be discovered in Papua New Guinea (Cámara-Leret et al., 2020). Although China has 2117 endemic threatened species, nearly 41% of them have not yet been documented (Chen et al., 2024), so the collection of new species is important for the conservation of these endangered species and the useful reference for assessing the conservation status of plant species (James et al., 2018). If there is a significant collection group bias, it can lead to an incomplete assessment of biodiversity and the management of conservation planning (Schmidt-Lebuhn et al., 2013; Cornwell et al., 2019). Therefore, future collection should expand the scope of plant specimens across diverse taxa, and encourage more botanists and plant enthusiasts to broaden the taxonomic preferences, identify underrepresented taxa and geographical gaps, and establish a more comprehensive, balanced specimen repository that accurately reflects Earth’s plant diversity thereby reducing taxonomic bias.

### 4.2 Collection data and geographical bias of new plant species

In this study, the peak number of newly discovered plant specimens was occurred in the 1920 - 1940, 1950 - 1960, 1970 - 1990 and 2000 - 2022. During the 1920 - 1940, the first group of plant taxonomists, the increased funding of botanical research from the government, and civil society groups contributed to the specimen collection and the new species discovery (Yang et al., 2013). The reason for the early peak (1950 - 1960) of collection is related to the compilation of the Flora Reipublicae Popularis Sinicae and the national scientific research resumed (Zhang et al., 2024b). During the time of 1958 to 1960 (i.e., the Great Leap Forward), many amateurs were encouraged to participate in plant specimens collections (Yang et al., 2013). Our study also supports the previous studies that the collection of botanical specimens is positively correlated with the social stability and prosperity (Panchen et al., 2019; Holopainen et al., 2012). The third peak (1970 - 1990) of the new species specimen collection might be influenced by national policies and economic development, for instance, large-scale scientific research activities (e.g., comprehensive scientific survey of the Qinghai-Tibet Plateau in 1973 - 1980 and comprehensive investigation in the Hengduan Mountains area in 1980 - 1985) (Yang et al., 2013). In the past two decades, the advanced technological developments of herbarium-making equipment and the improved transportation help to the new species collection, and the biodiversity protection policies push a series of plant biodiversity collection. For example, the fourth National survey of traditional Chinese medicine resources collected more than 1.5 million plant specimens and published nearly 200 new species. And about 60% of these new species have potential medicinal or traditional Chinese medicine effects (Huang et al., 2019). Another reason is that collecting and publishing descriptions of new species are temporally separated processes (Salle et al., 2009). Only a small number of species are identified at the time of collection, while the majority of new species remain unidentified for some time and are later described from herbarium specimens (Bebber et al., 2010). Besides, our study supports the high number of new species found in spring and summer, with the majority of plant specimens collected in July, and sampling during this time allows for the collection of plants in good flowering and fruiting condition. Previous collectors tended to regions with lower temperature of the warmest months and relatively high average monthly precipitation, because the superior water and heat conditions allow plants to thrive and provide ample water and food for livestock (Monsarrat et al., 2018). Digital specimen collections are also able to assist in the understanding of plant-pollinator interactions and the impacts of global change on plant diversity (Marín-Rodulfo et al., 2024). If collected only during specific months, it can lead to an incomplete assessment of the plant - pollinator relationship.

We found that the collection regions of new species were mostly in the southern provinces of China, concentrated between 20°N and 40°N, including Sichuan, Yunnan, Hubei and Zhejiang Province. The imbalance of sample collection at county level was particularly obvious, especially in Tianjin, Shanghai, Ningxia, Beijing, Jilin where these counties and cities found little or no new species. The reason is that the southern part of China has abundant water and heat resources, superior climatic conditions than the northern region, and there are large areas of mountainous regions. In mountainous regions, the habitat heterogeneity is higher, the plant diversity is richer, and it is easier to find new species (Zu et al., 2023b). In addition, research on new species in mountainous regions of China is mainly concentrated in the regions below 2000m above sea level because these lower elevations offer favorable hydrothermal conditions and fertile soils, which support a greater diversity of species (Wang et al., 2010). Recent research demonstrates that elevation significantly influences species diversity, with varying impacts across distinct vegetation strata. The shrub and herbaceous layers exhibit the most pronounced responses to elevational gradients, thereby directly affecting the detection rates of new species (Zhang et al., 2024b). Secondly, plant specimen collections in lower altitude regions are more accessible and benefits from convenient transportation, attracting the majority of botanical endeavors (Yang et al., 2014). The ecological niche breadth of species can influence the number of plant specimens collected. Species with narrow niches, geographically restricted, are less likely to be detected and thus resulting in fewer specimens (Inman et al., 2021). For example, Epiphytic orchids with small size, are restricted in distribution to three or fewer populations in a limited range. These traits resulting in tough challenges for identification and collection (Yang et al., 2020). Conversely, species with wide distribution and elevation ranges are easier to collect, leading to more new species discoveries (Yang et al., 2020). However, future plant specimen collection should extend beyond low altitudes, middle-to-low latitudes and areas characterized by high species richness. The scope of specimen collections should be expanded, and greater emphasis should be placed on plants at elevated altitudes and narrow niches. Using standardized methods and recording details, including site, time, elevation, plant type and habitat can identify specimens from high-elevation and narrow-niche areas, unlocking the potential for new species discoveries.

### 4.3 Collectors bias and the implications for future biodiversity conservation

The preferences and focus of collectors play a very important role in the discovery of new species. For instance, certain collectors may concentrate on species inhabiting specific environments, critically endangered species, or flora characterized by flowers and fruits (Panchen et al., 2019; Chen et al., 2012). In this study, we found that there is little difference between team and individual contributions to new species, but team contributions have gradually increased over the last two decades. The individual collectors were mainly botanists, taxonomists and individuals with rich botanical knowledge, while the team collectors were also mostly scientific expeditions composed of trained scientists and taxonomists and teams of university teachers and students. This study points out that the collection of plant specimens in the future will no longer rely solely on individualism, but will require the cooperation of multidisciplinary teams. Team collaboration with assigned roles enhances collection efficiency, while peer monitoring ensures strict adherence to sampling protocols, minimizing bias to yield reliable botanical specimens.

Botanical collectors play an important role in the digitization of plant specimens in China, the development of plant taxonomy, and future biodiversity conservation. Especially, their contribution is vital in the digitization process of plant image specimens captured in the field. Full digitization of type specimens globally can enhance collaboration among scientists worldwide, which is important for improving the efficiency of new species discovery and biodiversity conservation in the future (Orr et al., 2020). Recently, more and more citizen scientists are contributing to the discovery of new plant species, suggesting that the discovery of new species is no longer the exclusive domain of plant taxonomists, and that the public will become increasingly important in the discovery of new plant species in the future. For example, previous study integrated data sets from experts and volunteers on invasive species in parts of Wisconsin, revealing volunteers’ significant role in understanding species distribution, conducting sampling, analyzing environmental gradients, and assessing habitat suitability (Crall et al., 2015). This highlights the importance of citizen science in filling species distribution data gaps and expanding sampling coverage. The initial documentation of *Gastrodia pushparaga* (Orchidaceae), a newly described mycoheterotrophic orchid species discovered in Sri Lanka, originated from plant photographs inadvertently uploaded to iNaturalist by an ornithological observer (Bandara et al., 2020). This finding underscores the value of citizen science platforms in engaging enthusiasts across diverse disciplines and facilitating the serendipitous discovery of novel species. This study suggests that the efficient discovery of new species should rely on knowledge exchange between academia and the public and private sectors. In the future, artificial intelligence technologies such as mobile Internet and molecular biology technologies should be used to fully strengthen the cooperation between plant lovers and scientists, and make more contributions to the discovery of new species and biodiversity conservation (Kuzmina et al., 2017).

## Data Availability

The original contributions presented in the study are included in the article/**Supplementary Material**. Further inquiries can be directed to the corresponding authors.
