# Supplementary material for: How new plant species have been discovered in China: collection gaps and preferences over the past century
Source: Front Plant Sci. 2025 Jul 10;16:1605431. doi: 10.3389/fpls.2025.1605431 (PMC12287055; doi:10.3389/fpls.2025.1605431)
Supplement: Supplementary file 1 [file Supplementaryfile1.docx]

**Supporting information:**

**Title: How new plant species have been discovered in China: collection gaps and preferences over the past century**


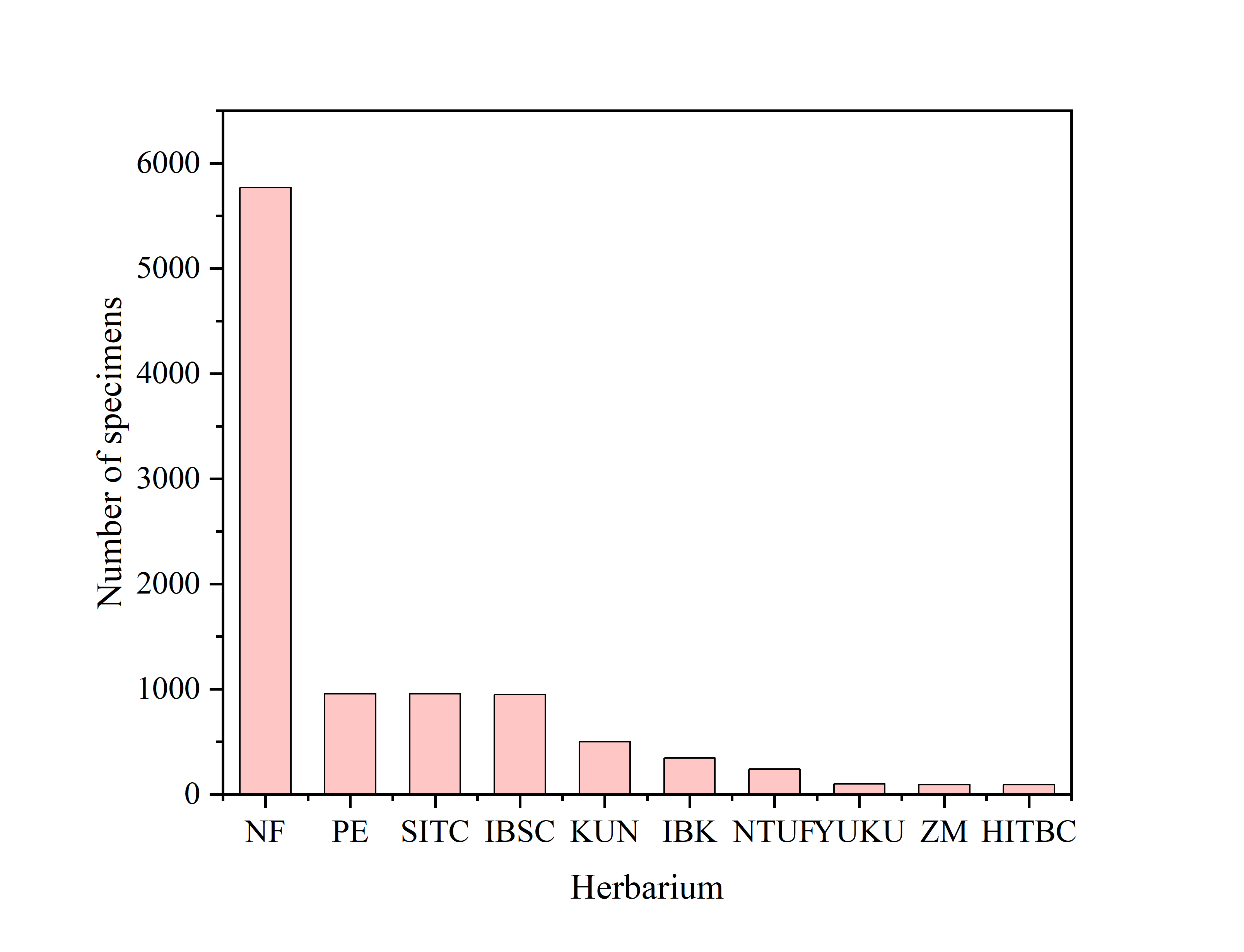


Figure S1 Discover the top ten herbarium in the number of plant specimens.


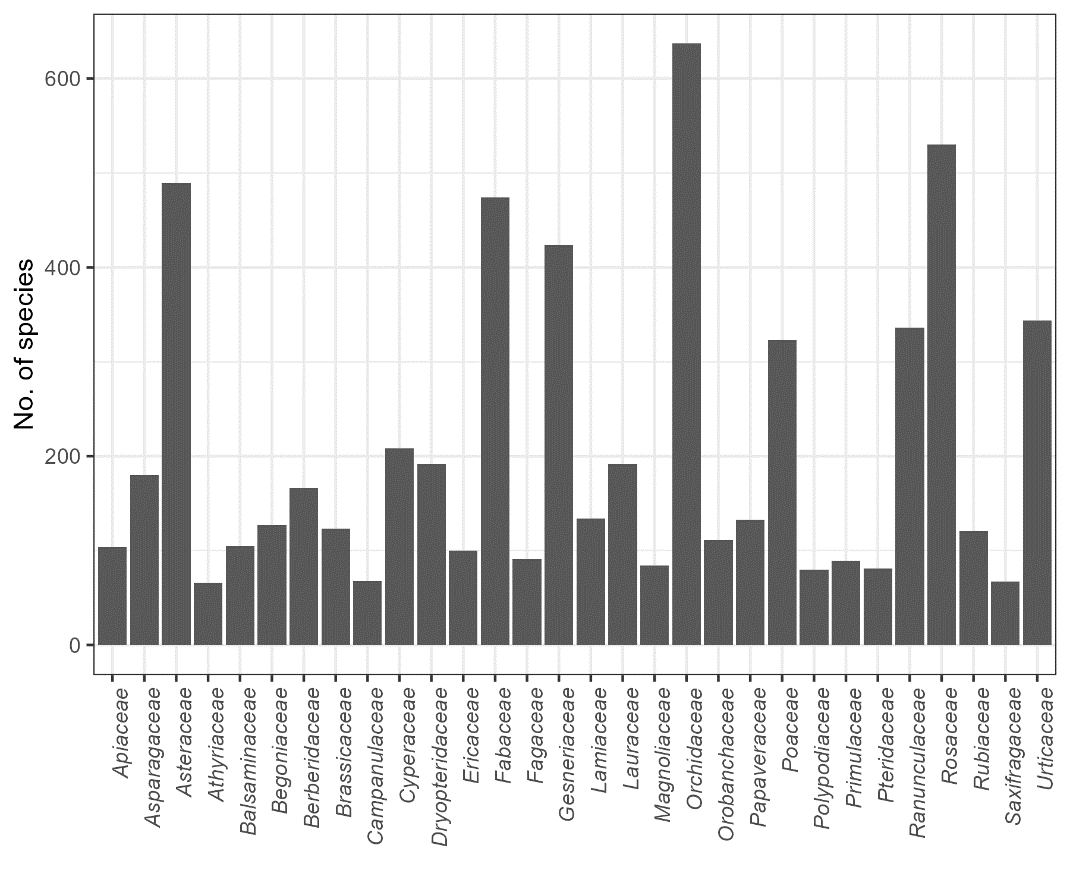


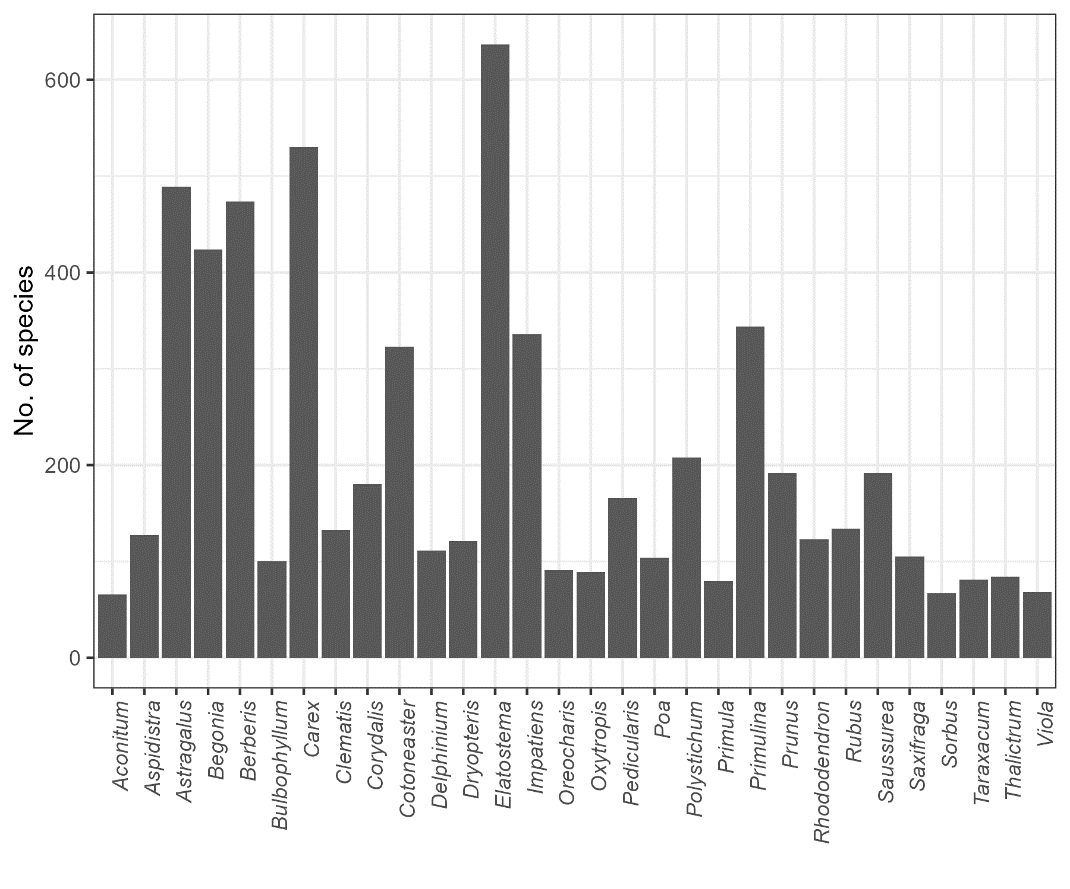


Figure S2 The top 30 families and genera with the largest number of new species. Red represents the number of specimens, blue represents the number of species.


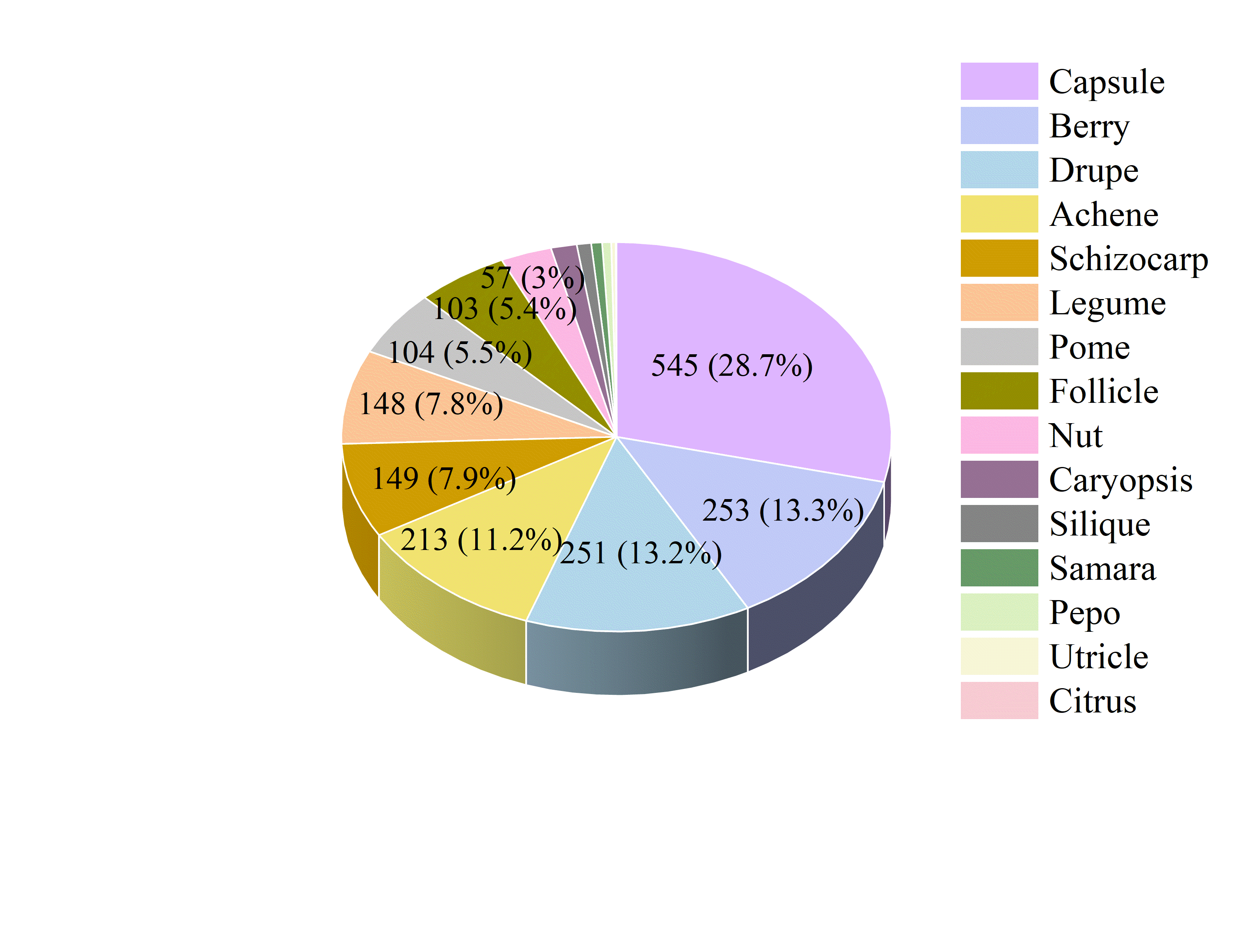


Figure S3 The proportion of newly discovered species with different fruit type.


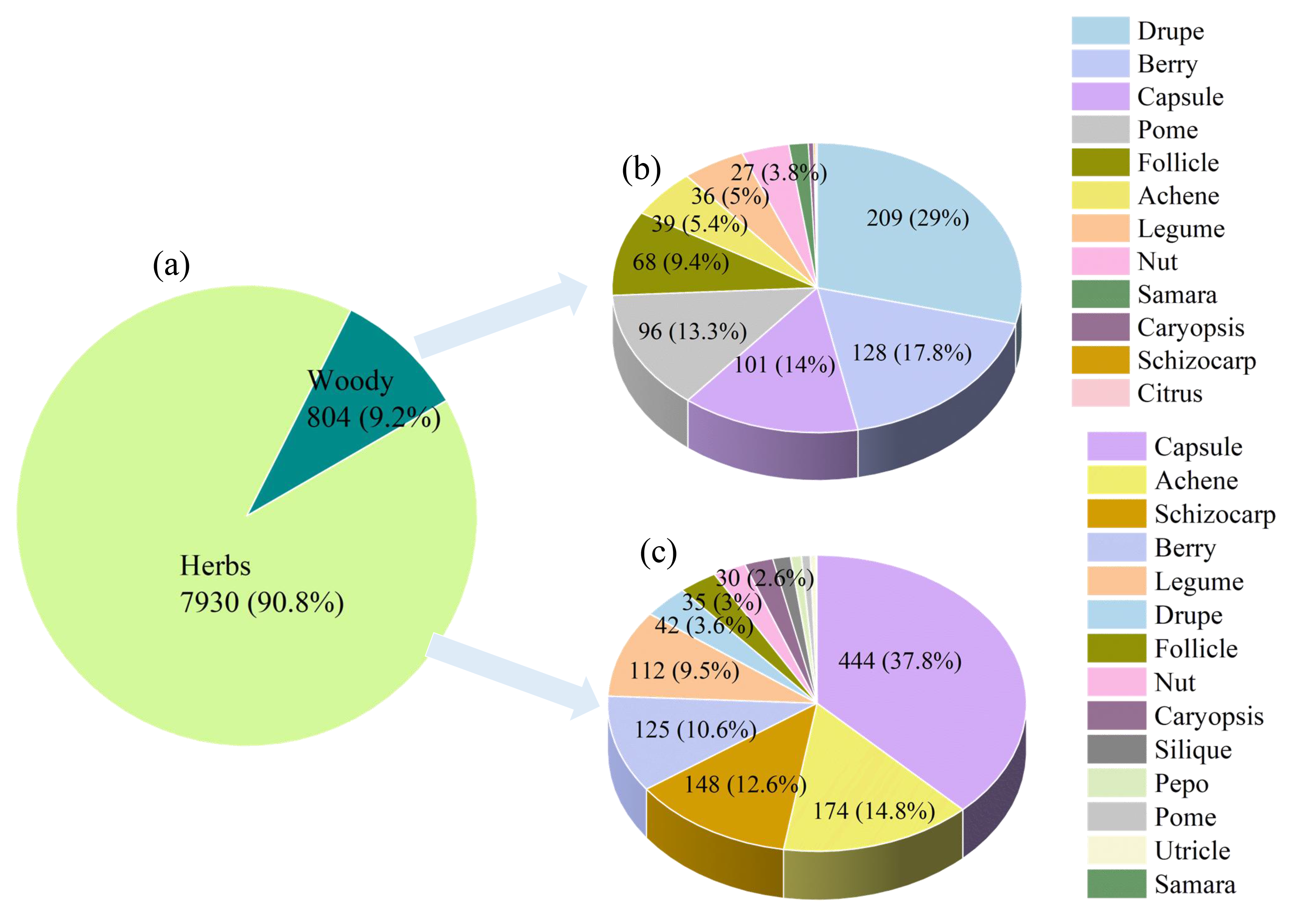


Figure S4 The proportion of newly discovered species with different fruit type in woody and herbs.

Table S1 Ranking of counties/cities where new species are found and the proportion of new species to total species.

| Counties/cities | Number of specimens | Number of species | Number of species /total species |
| --- | --- | --- | --- |
| Wenchuan | 374 | 219 | 2.51% |
| Nanchong | 229 | 150 | 1.72% |
| Nanjing | 328 | 124 | 1.42% |
| Motuo | 81 | 78 | 0.89% |
| Lichuan | 158 | 77 | 0.88% |
| Beibei | 112 | 70 | 0.80% |
| Libo | 100 | 59 | 0.68% |
| Deqin | 97 | 51 | 0.58% |
| Cangxi | 54 | 45 | 0.52% |
| Malipo | 44 | 44 | 0.50% |
| Hangzhou | 73 | 43 | 0.49% |
| Kangding | 55 | 42 | 0.48% |
| Gongshandulongzunuzu | 41 | 41 | 0.47% |
| Nanchuan | 52 | 38 | 0.44% |
| Dexing | 46 | 33 | 0.38% |
| Kunming | 36 | 30 | 0.34% |
| Pingbianmiaozu | 27 | 27 | 0.31% |
| Huaan | 41 | 27 | 0.31% |
| Hualian | 26 | 26 | 0.30% |
| Guangzhou | 30 | 26 | 0.30% |
| Chayu | 25 | 25 | 0.29% |
| Dali | 24 | 24 | 0.27% |
| Taizhong | 23 | 23 | 0.26% |
| Xichou | 22 | 22 | 0.25% |
| Qingdao | 32 | 22 | 0.25% |
| Linzhi | 25 | 22 | 0.25% |
| Daocheng | 26 | 22 | 0.25% |
| Nanping | 31 | 21 | 0.24% |
| Longshenggezu | 21 | 21 | 0.24% |
| Baoxing | 20 | 20 | 0.23% |
| Wan | 28 | 19 | 0.22% |
| Weixilisuzu | 19 | 19 | 0.22% |
| Taoyuan | 19 | 19 | 0.22% |
| Jinping | 19 | 19 | 0.22% |
| Jinghong | 19 | 19 | 0.22% |
| Yilan | 18 | 18 | 0.21% |
| Shennongjialin | 19 | 18 | 0.21% |
| Nielamu | 19 | 18 | 0.21% |
| Qianshan | 31 | 17 | 0.19% |
| Suichang | 19 | 17 | 0.19% |
| Maguan | 17 | 17 | 0.19% |
| Hekouyaozu | 17 | 17 | 0.19% |
| Leshan | 21 | 17 | 0.19% |
| Wuhan | 21 | 16 | 0.18% |
| Shangsi | 16 | 16 | 0.18% |
| Napo | 16 | 16 | 0.18% |
| Jingdongyizu | 16 | 16 | 0.18% |
| Longzhou | 16 | 16 | 0.18% |
| Yongan | 21 | 15 | 0.17% |
| Mulizangzu | 15 | 15 | 0.17% |
| Kangding | 15 | 15 | 0.17% |
| Emei | 15 | 15 | 0.17% |
| Bomi | 16 | 15 | 0.17% |
| Daocheng | 17 | 15 | 0.17% |
| Zhenkang | 14 | 14 | 0.16% |
| Menghai | 14 | 14 | 0.16% |
| Leibo | 15 | 14 | 0.16% |
| Funing | 14 | 14 | 0.16% |
| Chengdu | 19 | 14 | 0.16% |
| Yadong | 13 | 13 | 0.15% |
| Pingshan | 14 | 13 | 0.15% |
| Linan | 16 | 13 | 0.15% |
| Lijiang | 14 | 13 | 0.15% |
| Huizhou | 18 | 13 | 0.15% |
| Emeishan | 13 | 13 | 0.15% |
| Zhenjiang | 14 | 12 | 0.14% |
| Nanchuan | 12 | 12 | 0.14% |
| Tianquan | 14 | 12 | 0.14% |
| Pingxiang | 25 | 12 | 0.14% |
| Jilong | 13 | 12 | 0.14% |
| Fuzhou | 16 | 12 | 0.14% |
| Xianggelila | 11 | 11 | 0.13% |
| Yizhang | 16 | 11 | 0.13% |
| Xiangcheng | 13 | 11 | 0.13% |
| Xinbei | 11 | 11 | 0.13% |
| Simao | 12 | 11 | 0.13% |
| Nantou | 11 | 11 | 0.13% |
| Heqing | 11 | 11 | 0.13% |
| Luding | 11 | 11 | 0.13% |
| Jiulong | 11 | 11 | 0.13% |
| Chengkou | 12 | 11 | 0.13% |
| Chengbumiaozu | 13 | 11 | 0.13% |
| Wen | 10 | 10 | 0.11% |
| Yingjiang | 10 | 10 | 0.11% |
| Xishuangbannaidaizu | 10 | 10 | 0.11% |
| Xuyi | 12 | 10 | 0.11% |
| Milin | 11 | 10 | 0.11% |
| Sanya | 10 | 10 | 0.11% |
| Ruyuanyaozu | 10 | 10 | 0.11% |
| Qingyuan | 10 | 10 | 0.11% |
| Longquan | 11 | 10 | 0.11% |
| Jinxiuyaozu | 10 | 10 | 0.11% |
| Guan | 11 | 10 | 0.11% |
| Maerkang | 10 | 9 | 0.10% |
| Huanjiangmaonanzu | 9 | 9 | 0.10% |
| Fugong | 9 | 9 | 0.10% |
| Badong | 9 | 9 | 0.10% |
| Yangbiyizu | 8 | 8 | 0.09% |
| Yongxing | 8 | 8 | 0.09% |
| Yixing | 9 | 8 | 0.09% |
| Zunyi | 9 | 8 | 0.09% |
| Shuangpai | 8 | 8 | 0.09% |
| Taishun | 8 | 8 | 0.09% |
| Nanping | 14 | 8 | 0.09% |
| Lishui | 14 | 8 | 0.09% |
| Leshan | 8 | 8 | 0.09% |
| Longling | 8 | 8 | 0.09% |
| Jiujiang | 10 | 8 | 0.09% |
| Jingningshezu | 8 | 8 | 0.09% |
| Ji | 11 | 8 | 0.09% |
| Lasa | 10 | 8 | 0.09% |
| Jurong | 8 | 8 | 0.09% |
| Dingjie | 8 | 8 | 0.09% |
| Baotinglizumiaozu | 8 | 8 | 0.09% |
